# Deep-SAGA: a deep-learning-based system for automatic gaze annotation from eye-tracking data

**DOI:** 10.3758/s13428-022-01833-4

**Published:** 2022-06-01

**Authors:** Oliver Deane, Eszter Toth, Sang-Hoon Yeo

**Affiliations:** 1 School of Sport, Exercise and Rehabilitation Sciences, The University of Birmingham, Edgbaston, Birmingham, B15 2TT UK; 2 School of Psychology, The University of Birmingham, Birmingham, UK

**Keywords:** Gaze tracking, Portable eye-tracker, Object detection, Deep learning, Masked region-based convolutional neural network

## Abstract

With continued advancements in portable eye-tracker technology liberating experimenters from the restraints of artificial laboratory designs, research can now collect gaze data from real-world, natural navigation. However, the field lacks a robust method for achieving this, as past approaches relied upon the time-consuming manual annotation of eye-tracking data, while previous attempts at automation lack the necessary versatility for in-the-wild navigation trials consisting of complex and dynamic scenes. Here, we propose a system capable of informing researchers of where and what a user’s gaze is focused upon at any one time. The system achieves this by first running footage recorded on a head-mounted camera through a deep-learning-based object detection algorithm called Masked Region-based Convolutional Neural Network (Mask R-CNN). The algorithm’s output is combined with frame-by-frame gaze coordinates measured by an eye-tracking device synchronized with the head-mounted camera to detect and annotate, without any manual intervention, what a user looked at for each frame of the provided footage. The effectiveness of the presented methodology was legitimized by a comparison between the system output and that of manual coders. High levels of agreement between the two validated the system as a preferable data collection technique as it was capable of processing data at a significantly faster rate than its human counterpart. Support for the system’s practicality was then further demonstrated via a case study exploring the mediatory effects of gaze behaviors on an environment-driven attentional bias.

## Introduction

With visual acuity diminishing in the eye’s periphery, adaptive eye movements ensure that the high-resolution fovea prioritizes visually informative regions of any presented scene (Findlay & Gilchrist, 2003). As such, one’s ability to understand the visual world is heavily dependent on these eye movements sampling a scene and guiding visual attention towards worthy targets for perception. A vast body of research is committed to determining how this gaze distribution occurs during natural navigation and locomotion, as any contribution to addressing the issue has significant implications across disciplines (e.g., Davoudian & Raynham, 2012; Kretch & Adolph, 2015; Trefzger et al., 2018).

While previous studies investigating gaze behaviors relied mainly on artificial laboratory designs, recent advances in minimally intrusive, portable eye-tracking glasses with forward-facing scene cameras have legitimized the option of collecting data from entirely naturalistic experimental conditions. The benefits of high ecological validity and generalizability suggest that these mobile alternatives should be utilized when exploring in-the-wild gaze behaviors. This proposition is supported by studies reporting behavioral discrepancies between gaze data collected in the lab using static trackers to that obtained in real-world trials with portable alternatives (Foulsham et al., 2011; Foulsham & Underwood, 2008; Hayhoe et al., 2003; Marius't Hart et al., 2009).

However, the advanced mobility offered by portable trackers introduces new impediments regarding data annotation and analysis. Without the benefit of predetermined visual stimuli appearing in a fixed two-dimensional screen, and with the added complexity of participants encountering uncontrolled, spontaneous, and dynamic visual experiences, identifying which stimuli and regions are gazed upon at each point of time poses a significant challenge when using portable devices. A conventional solution for this relies on a manual coding approach: a human encoder compares, frame-by-frame, the eye-tracking data and the corresponding video image recorded in the scene camera. By comparing these two sets of information, the human encoder then analyzes where and on what object the participant's gaze fell during navigation (Foulsham et al., 2011; Trefzger et al., 2018; Zult et al., 2019). While thorough, this approach is not scalable and only applicable to short video footages (up to 30 seconds in the case of Foulsham et al., 2011) since dealing with large data sets is unfeasibly time-consuming.

Several solutions have been proposed for automating annotation, thus reducing workload and maximizing data set size. For example, Olsen (Olsen, 2012) proposed a system combining a mobile eye-tracker and infrared markers. These markers are physically placed in navigation environments and act as reference points allowing gaze localization. By placing objects in fixed, preregistered locations with respect to the markers, their proposed system can estimate which objects were gazed upon in much the same way as original screen-based designs (Evans et al., 2012). However, such methods only work on the premise that markers are deployed in environments and objects stay in predefined locations at all times, and therefore their application scope is limited to highly controlled navigation scenarios.

In addition, studies have proposed systems incorporating conventional object recognition algorithms (ORAs) for automatic data annotation (De Beugher et al., 2012; Toyama et al., 2012). For frame-by-frame images captured by a scene camera, these systems automatically extract the region around the gaze location and compare its contents to the images of the preregistered object shapes using conventional feature-matching algorithms such as scale-invariant feature transform (SIFT) (Lowe, 1999). This approach can automatically inform when relevant objects are gazed upon without requiring designated markers or prescribed object positions. However, while effective on an ad hoc basis when exploring gaze behaviors towards specific preregistered objects such as museum exhibits (Toyama et al., 2012), these systems lack flexibility as conventional ORAs are essentially shape-matching algorithms that work only for preregistered objects with fixed shapes. Since the recognizable objects must exactly match their pre-specified shapes, these systems cannot handle object types with inter-object (e.g., cars and buildings) or intra-object variances (e.g., humans and animals). Notably, efforts have been made to include variable shapes such as human faces and bodies (De Beugher et al., 2014) but the flexibility of such a system is not easily generalizable since it requires a set of designated ORAs, each tailored for a specific object type.

Markedly, these limitations are mainly due to (1) restraints in available computing power and (2) an absence of sufficiently robust computer vision algorithms for object detection and recognition. Advancements in computing power, fueled by developments in accessible graphics processing units (GPUs), together with the recent revolution in deep learning (DL) that has seen the introduction of increasingly robust and efficient object detection networks, have provided a means to overcome these bottlenecks. While recent studies have applied DL-based computer vision algorithms for psychological experiments including, for example, human-to-human interactions (Callemein et al., 2018), no study, to the best of our knowledge, has deployed this technology to explore the automation of in-the-wild gaze data annotation. Indeed, the lack of a general-purpose annotation tool for in-the-wild scenarios is evidenced by the fact that recent papers exploring natural navigation remain reliant on the manual coding process outlined earlier in this introduction (Trefzger et al., 2018; Zult et al., 2019). Therefore, the current study attempts to fill this gap by proposing a general-purpose data analysis tool that harnesses the current state-of-the-art in DL networks to capably automate the annotation process for gaze footage recorded in real-world conditions.

**Fig. 1:**
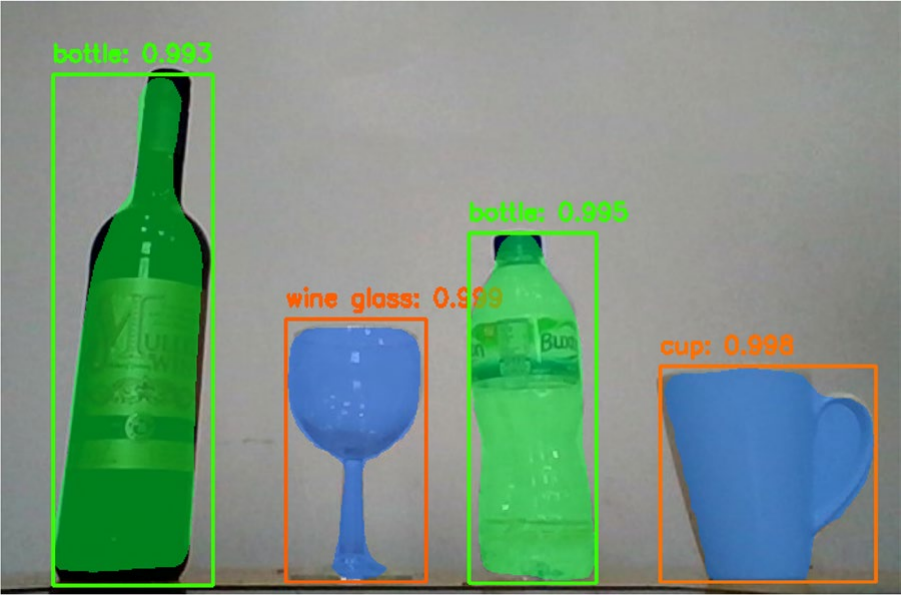
Example of object recognition and segmentation using Mask R-CNN. For each recognized object, the network outputs a bounding box (a colored box surrounding objects), object type (texts on the top left corner of the bounding box), and a binary mask (a colored region overlaying the object) indicating pixels within the bounding box that are occupied by the object

The proposed method combines eye-tracking data with the output of an object detection and instance segmentation algorithm: the Masked Region-based Convolutional Neural Network (Mask R-CNN) (He et al., 2017). For each scene image, the Mask R-CNN automatically recognizes and labels objects, and outputs the corresponding “masks” segmenting regions in pixels that are occupied by each object (see “The masked regional convolutional neural network” and Fig. 1). By feeding it the scene video recorded by a head-mounted camera and combining its output with frame-by-frame gaze coordinates captured by the eye-tracking glasses, the proposed system offers accurate frame-by-frame information on what objects in which locations were gazed upon during navigation.

For the rest of this paper, we describe the technical details of the proposed framework in “Methods: System development” and provide results from a performance evaluation comparing the output of the system to that of manual coders in “System evaluation”. In “Case study”, we present an exploratory case study where our system was integrated into an existing experimental paradigm investigating the environmental effect on attentional bias. We will demonstrate how the result of the experiment can be reinterpreted or reinforced based on the richer information and analytical power offered by the proposed system. Discussions on limitations and future directions will be presented in “Conclusion and discussion”.

### Methods: System development

As mentioned above, the proposed system integrates two methods: (1) portable binocular eye-tracking glasses with a built-in scene camera (Kassner et al., 2014) and (2) Mask R-CNN: a DL-based video image processing algorithm capable of automatically recognizing and locating objects (He et al., 2017). By integrating the output of the two for each frame of the video recorded by the scene camera, the system can obtain combined information of location and the type of object the subject is gazing at without any manual coding.

### Eye-tracking device

We used commercially available portable binocular eye-tracker glasses with a built-in scene camera (Pupil Core, Pupil Labs GmbH). During navigation, a scene video (100° diagonal field of view) was recorded at 30 Hz, and the position and shape of pupils were tracked at 200 Hz using two near-infrared eye cameras, with accuracy and precision of 0.60° and 0.02, respectively. Combining these, the gaze position in screen coordinates was estimated using a built-in algorithm based on a nine-point calibration method (see “Case Study Methods” for details) and automatic drift compensation.

### The masked regional convolutional neural network

The Mask R-CNN is the current state-of-the-art DL-based computer vision algorithm for instance segmentation. Put simply, Mask R-CNN can recognize objects in a scene, and with the recognized type and the location of the object, the network performs instance segmentation, a procedure of computing a binary mask that outlines the object’s shape in space (Fig. 1). The framework deploys a traditional convolutional neural network, a prevalent DL architecture for object recognition, and combines this with a “Region-of-Interest Align” module which permits the computation of regional information of the recognized objects. The proposed system imported an open-source Mask R-CNN developed by Abdulla (2017), which was pre-trained with the Microsoft Common Objects in Context (MS COCO) data set (Lin et al., 2014) and able to detect and segment 91 different object types. Importantly, the network is highly robust and flexible in recognizing objects in the same category but in different shapes, mainly because such DL-based object recognition methods are generally based on massive data sets. For instance, the 91 object categories recognizable in our Mask R-CNN network were trained based on 200,000 labeled images and 1.5 million object instances, including 250,000 human images, provided by the MS COCO data set (Lin et al., 2014), which indicates that the network can deal with huge intra-categorical variabilities. The network was run on an NVIDIA Tesla K80 GPU via Google Colaboratory.

### The complete gaze detection system

After calibration (see “Case study methods” for details), the gaze detection process begins with users navigating a route while wearing the eye-tracker device. Upon completion, the video footage obtained by the scene camera is split into frames, and each frame is registered with accompanying gaze information represented in screen coordinates (1280-by-720 from the upper-left corner). Video frames are then fed to the Mask R-CNN for object recognition and masking. Network output for each frame consists of a set of mask–label pairs, where the number of pairs corresponds to the number of objects recognized. Each pair consists of a mask, 1280-by-720 binary matrix indicating whether each pixel is occupied by that object or not, and a label specifying object type.

**Fig. 2:**
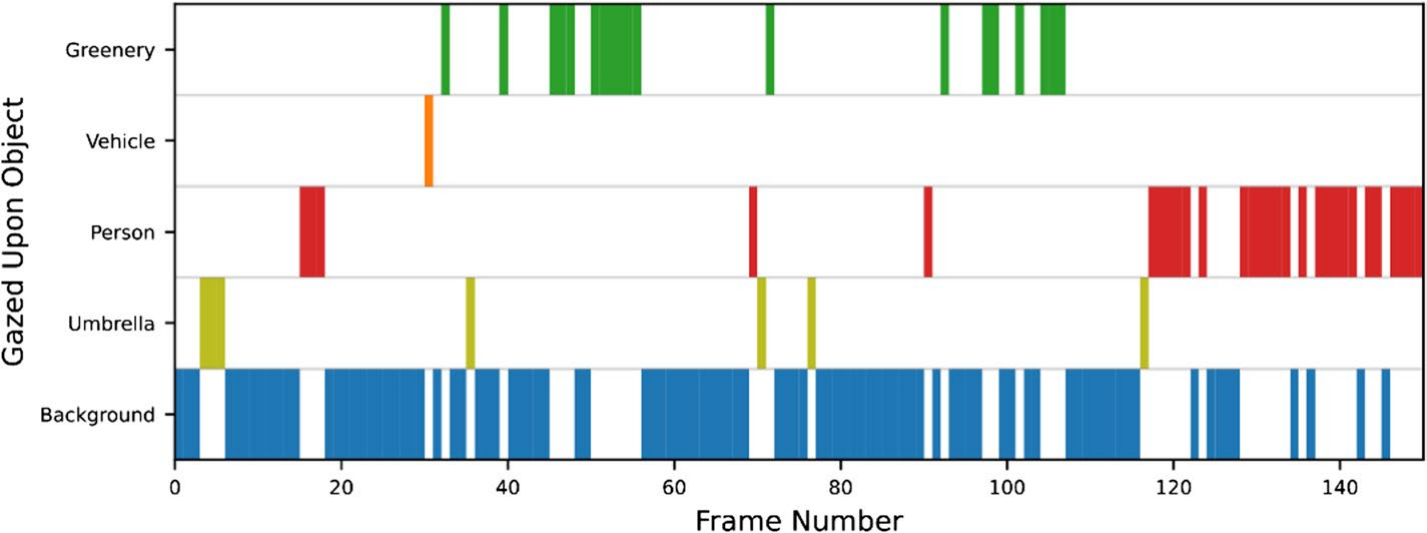
A visualization of the system’s final output that outlines what is looked at for each frame of footage

These localization outputs for each object were then sequentially compared to the captured gaze location represented in the screen coordinates, abbreviated as “gaze coordinate.” If a gaze coordinate overlaps with a mask, then the corresponding object was regarded as being gazed upon and was added to a “gazed upon” list (GU-list). Note that a gaze point is considered to be on an object when it falls upon its masked pixel, rather than within its bounding box in order to ensure precise estimates. As such, the completed GU-list would outline which object was being looked at for every frame of input footage, as illustrated in Fig. 2.

In addition to the object-based detection by Mask R-CNN, the proposed system is also able to include non-object-based masking algorithms. As an example, for the case study presented in “Conclusion and discussion”﻿, which explores the psychological effect of being in a natural green environment, our system incorporates a color-based masking algorithm since the capability of Mask R-CNN is relatively limited in recognizing various natural features, e.g., grassy areas, bushes, trees. To achieve this, each frame is first converted from RGB format to hue-saturation-value (HSV) color space (Sural et al., 2002) where hue (H) value defines pixel color mapped from red (0) to purple (360). Based on this, a group of pixels whose H values are between 121 and 180 was defined as a “Greenery” mask. As was done for object-based masks, if gaze overlaps with this mask, a Greenery label was appended to the GU-list. To ensure that artificial objects, such as green cars or green clothes, were not unintentionally labeled as Greenery, this check was only conducted when no identified object was being gazed at. Importantly, when deployed during real experiments, lighting conditions will vary substantially across different input videos. This would be particularly evident in the case of footage captured during outside, in-the-wild navigation. As a result, fixed greenery threshold values would result in significant levels of greenery detection errors. To counter this, the system provides an option whereby users can observe the greenery output (i.e., a processed image containing the greenery overlay) for a random subsample of video frames *before* processing the entire video. These sample frames would be initially processed with default greenery values—the HSV values that define the color threshold used by the detector to classify areas of the image as greenery or not. Having observed the output, users can then fine-tune this value until the greenery detector is identifying green areas with optimal accuracy.

Together with the GU-list, the system delivers a series of behavioral parameters, named *scene variables*, that summarize objects that appeared and the corresponding gaze behavior. The first, which we term *saliency*, presents the percentage of frames where a gaze fell upon each object. The second, *frequency*, concerns the percentage of frames, out of all frames, where an object appeared during navigation, irrespective of whether the item was gazed upon by the participant. For the Greenery feature, these frequency scores are obtained by taking the average percentage of greenery pixels, out of the total number of pixels, in each frame. A final *persistency* parameter captures temporal information, reporting the length of fixations when individual objects were gazed upon. Only persistency upon discernable objects was computed, while frames with a Background, Other, or Greenery label were discarded. In addition, to prevent accidental detections, persistency is only computed when the dwell time of gaze is longer than 200 ms (this corresponds to six consecutive frames in 30 Hz videos), which is the generally accepted minimal duration for gaze fixations (Salvucci & Goldberg, 2000).

Regarding the computation of the persistency, it should be noted that Mask R-CNN is not capable of object tracking, and therefore it does not provide any information on whether two objects of the same type shown in two adjacent frames are the same object or not. While the persistency can be easily computed when the gaze is switching among different objects with different labels, the absence of tracking makes it challenging to compute the persistency when the gaze is switching among multiple objects of the same label (e.g., looking at different people in a crowd). To deal with this, the proposed system uses the following simple heuristic algorithm determining, in between two adjacent frames, whether the gaze is fixated on the same object or is moved to a different object of the same type: A fixation on an object is deemed to have ended when the gaze coordinates of the current frame deviated more than 5.2° from that of the previous. This threshold value of 5.2° is the mean saccade magnitude for scene perception measured by Rayner et al. (2007). As the study was based on a small screen (19-inch screen located 60 centimeters away), the mean magnitude reported in their study was used as the minimum saccade magnitude in our system. The accuracy of this heuristic algorithm, compared to human coders, will be analyzed in “Evaluation results”, and its limitations and future solutions will be discussed in “Limitations and future work”.

**Fig. 3:**
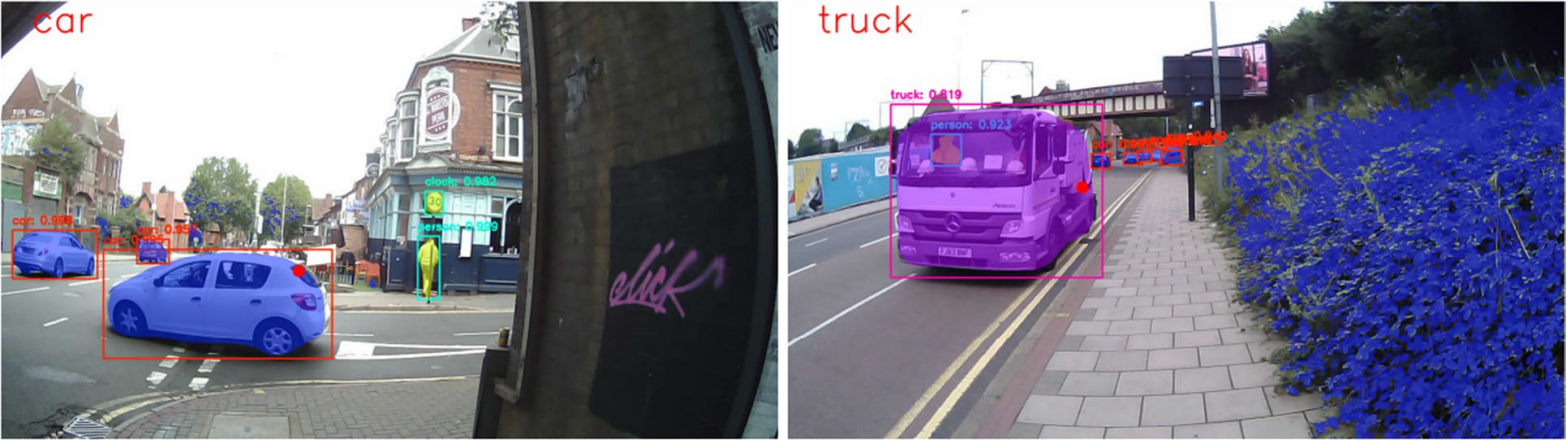
Screenshots of the system’s output video complete with greenery masks (blue), labeled objects, gaze overlay, and “gazed upon” object label. An example of video footage can be found in Yeo (2020)

To enable the visualization of the above processes, the system wrote all frames to a final video at the same frame rate as the input video (30 fps). This contained the output of the Mask R-CNN (complete with bounding boxes, binary masks, and corresponding object labels), as well as the computed greenery mask. The system also overlaid a circle representing gaze location and a label at the top of the screen that depicted what was currently being gazed upon (Fig. 3). When the gaze fell upon neither a recognizable item nor a greenery area, the corresponding label was set to Background.

## System evaluation

The performance of the proposed system was evaluated through comparison with naïve human coders. Both human coders and the proposed system annotated all frames in short video clips randomly taken from the entire video footage recorded for the case study to be presented in “Case study”. Based on the assumption that the annotation statistics of the recruited human coders are representative of human baseline performance, the analysis was focused on assessing the similarities and differences of the automatic annotation of the proposed system compared to the human performance.

### Evaluation methods

#### Participants

A total of seven participants (three female) were recruited via volunteer sampling. All were aged between 18 and 28 years and reported having normal or corrected-to-normal vision. In addition, all participants had at least a low-level understanding of the software used for manual annotations (Microsoft Excel). Each participant gave full informed consent to take part in the study. The experiment consisted of a single online session.

#### Materials

**Fig. 4:**
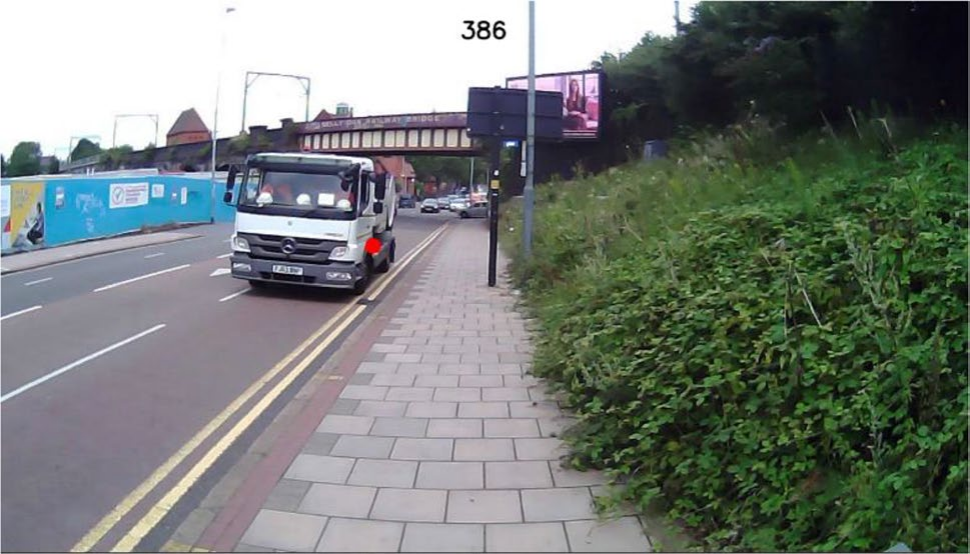
Example of an image provided to human coders for annotation, consisting of a video frame, frame number (top center), and gaze cursor overlay (red circle)

Frames were presented to participants via a PDF file containing still images of all frames within a given piece of footage. Each image was overlaid with both the gaze cursor, shown as a red circular mark, and the corresponding frame number (Fig. 4). Responses for each frame were contained within an Excel worksheet that followed a predefined template provided by researchers.

#### Procedure

Participants were divided into two groups (Group 1 with four coders and Group 2 with three) and each group was asked to annotate the same three 20-second video clips that are randomly sampled from the entire set of videos. The order in which videos were coded was counterbalanced across participants with the same group. Before commencing the coding process, each coder first partook in a 15-minute video call in which a member of the research team briefed them on the methodology to follow. They were then sent the necessary resources (PDF and Excel files for each of the three videos) to complete the coding process in their own time. The instructions presented to participants were as follows: They were first asked to open the Excel template file and the PDF document of their first video clip. Participants were instructed to open files in such a way that both Excel and PDF files were visible simultaneously.

The provided Excel file contained four columns: “Frame Index,” “Object Gazed Upon,” “Not sure?” and “New Item?”. The first column was filled with frame indices from 1 to the total number of frames of the video clip, and, for each frame, participants were instructed to fill in the remaining three columns. Within the “Object Gazed Upon” column, participants were instructed to write down the object being viewed upon in the given frame; thus, this column corresponds to the GU-list produced by the proposed system. Importantly, while the object labels recognizable by the proposed system are limited to 91 labels from the training data set (MS COCO data set), no specific guidelines were provided which could limit the object categories and specific names participants could use for annotation. Instead, they were explicitly asked to assign an appropriate label on their own judgment, except for an instruction to use Greenery labels for all greeneries. This is to ensure a more efficient coding procedure, and to allow a fair assessment of the system’s output quality by comparing it with unprocessed outputs from human coders.

If participants recognize that the gaze cursor was not on any discernible object, they were asked to write “Background.” Within the “Not Sure?” column, participants were asked to insert 1 (or leave it empty otherwise) when they find it difficult to tell which object was gazed upon. Finally, to annotate events where the gaze was switched to a different object of the same label, participants were instructed to insert 1 in the “New Item?” column (or leave it empty otherwise). This will be later compared with the result of the heuristic algorithm used by the proposed system to annotate these events.

Once comfortable with the procedure, participants coded the first frame, completing all three of the required fields, before clicking the right arrow key to progress to the next page of the PDF file displaying the second frame of the footage, and again completing the required fields for the corresponding row. Following this procedure, each participant produced a completed Excel spreadsheet, i.e., the four columns of annotation data for all frames, for each of three video clips assigned to them. These were then compared with the output of the proposed system.

#### Data processing and analysis

Since no constraint was put to the labels the human coders can use, comparisons across human coders, and between human coders and the system, require the following manual preprocessing: Labels that are different but are apparently referring to the same object type were grouped together. For example, labels such as “Sign” and “Road Sign” were merged into the “Sign Post” label. Labels referring to objects listed in the training data set used the data set’s term, while the remainder were assigned the most commonly used label amongst the combined coder output. All cases of preprocessed labels and their numbers of occurrence are summarized in “Evaluation results”.

After the preprocessing, the similarity of human annotations was assessed via cross-coder similarity scores computed among the GU-lists produced by human coders on the same video clip. The cross-coder similarity was evaluated using Fleiss’ Kappa (Fleiss & Cohen, 1973), a widely used measure for agreement on categorical ratings of more than two coders.

After analyzing the cross-coder similarity, the main analysis was focused on assessing the similarity between the GU-lists produced by each human coder and that by the proposed system. The similarity between the two GU-lists was measured by a weighted average F1-score, a common metric for evaluating classification performance. The weight for averaging is determined by each label’s support, i.e., the actual occurrence of the label in the data set (similar to the frequency in the proposed system). These weighted averages were used to adequately evaluate the system’s classification performance by considering the class imbalance normally observed in the data set, where Background accounted for 48.44% of all labeled frames and Greenery for 13.64%. For a fair comparison, care was taken to assess the impact of limiting labels to those recognizable by the system: For the human-coded labels that are not included in the set of labels recognizable by the system, similarity scores before and after their exclusion were both reported, in which the unrecognizable labels were all relabeled to Other after exclusion.

### Evaluation results

First, it was found that all participants annotated with low uncertainty, as indicated by the overall low occurrence (4.44%) of the “Not Sure” flag. The outcome of the preprocessing described in the previous section (“Evaluation methods”) is presented in Table 1. The first (Table 1) summarizes the labels in human coders’ annotations that are grouped together by preprocessing. As can be seen in the list of original labels, the ambiguity among human coders in using different labels was low and ordinary. Table 1 lists labels that are not included in the list of labels recognizable by the proposed system (and therefore relabeled as “Other”), with their number of instances in all GU-lists. Compared to the total number of frames, it was found that all these instances occupy small portions of the data (4.37%). These altogether suggest that experimental uncertainties—either by participant’s inexperience or by ambiguity in the provided instructions and images—and the corresponding preprocessing procedure had minimal effects on the main results.

**Table 1:** Preprocessing result. (A) The original labels assigned by human coders, with the number of times they appeared in the coder output totaled across coders, and the label assigned according to the most common term used by all coders. Labels in bold font are those recognizable by the proposed system. (B) The total number of object labels that did not appear in the recognizable labels of the proposed system, and therefore were relabeled to “Other”

A)		
**Original label**	**Total number of instances** ***(out of total 10,629 labeled frames)***	**Assigned label**
Lorry	66	Truck
Motorbike	5	Motorcycle
Van	485	Car
Human	9	Person
a-board	5	Advertising board
advert board	6	Advertising board
Sign	104	Sign post
Road sign	45	Sign post
Pole	21	Lamp post
Lamppost	68	Lamp post
Post	4	Lamp post
-	Total occurrences: 818 (7.70%)	-
B)		
**Grouped label**	**Total number of instances** ***(out of total 10,629 labeled frames)***	**Assigned label**
Sign post	150	Other
Bridge	91	
Lamp post	48	
Bin	54	
Bag	18	
Advertising board	55	
Bollard	9	
Square planter	5	
Vegetable stand	17	
Curb	17	
-	*Total occurrences: 464 (4.37%)*	*-*

After preprocessing, analysis was focused on the cross-coder similarity using Fleiss’ Kappa. The result shows that annotations by different participants were highly similar, as indicated by high cross-coder similarity scores for all videos: Fleiss’ Kappa averaged across videos was 0.881 with SD = 0.053. This observed strong agreement among human coders suggests that the effect of cross-coder variability on the results is limited.

**Fig. 5:**
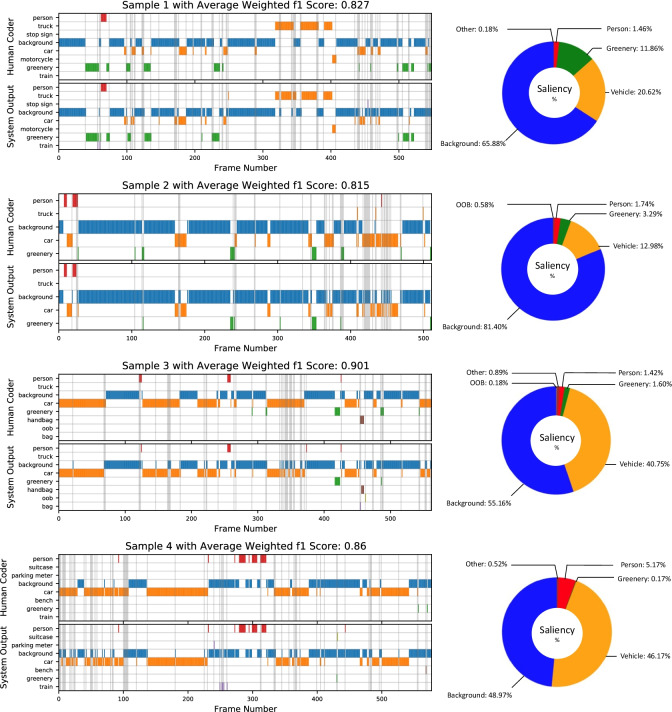
Left: comparison of the analysis results of the proposed system and one naïve human coder (selected at random) for four of the six sampled footages (Samples 1 to 4). Each row represents the type of object gazed at, where the “out of bound” (OOB) label indicates the frame where the gaze is out of the measurable region of the eye-tracker. Semi-transparent grey vertical lines highlight discrepancies between the two coders. The corresponding weighted average F1-score is displayed at the top of each sample. Right: pie charts displaying a breakdown of the percentage of frames in which each feature was gazed upon, analyzed by our system. For the sake of clarity, any instance of a Truck, Car, Bus, or Motorcycle was reassigned a Vehicle label for this latter visualization, while rare objects were given a general label Other﻿

As the main result, comparisons between human coders and the proposed system are presented in Table 2. Overall high weighted average F1-scores across all videos and also low variabilities (as indicated by small SD) across human coders suggest that there is a strong agreement between the proposed system and human coders. Frame-by-frame comparisons of the output of the proposed system to that of a single human coder are illustrated in Fig. 5 for four different video samples.

**Table 2:** The agreement scores, as measured by weighted average F1-scores, between human coder and system output. Scores for each of Groups 1 and 2 are averaged across outputs and human coders, respectively. Results from the unconstrained condition are presented in the middle column, and the right column shows scores following the exclusion of objects not included in the recognizable labels of the system

	Video number	Weighted average F1-scores (unconstrained labels)	Weighted average F1-scores (constrained labels)
Group 1 (n=4)	1	0.779 (SD = 0.021)	0.827 (SD =0.014)
	2	0.700 (SD = 0.023)	0.815 (SD =0.012)
	3	0.884 (SD = 0.015)	0.901 (SD =0.003)
Group 2 (n=3)	4	0.859 (SD = 0.031)	0.860 (SD =0.015)
	5	0.850 (SD = 0.044)	0.906 (SD =0.018)
	6	0.782 (SD = 0.021)	0.816 (SD = 0.025)
Mean	-	0.809 (SD = 0.068)	0.854 (SD = 0.041)

Among the frames in which outputs from the two coding methods disagreed, a proportion was due to a gaze falling upon items not included in the set of objects recognizable by the system (i.e., object labels not included in the data set that the system was trained on). This is highlighted by the higher average F1-scores apparent when the analysis only considered frames in which the human-reported gaze fell upon a trained object (0.851 for constrained labels compared to 0.809 for unconstrained labels). This is most apparent in video 2, where the system’s inability to recognize two salient objects (sign post and advertising board) hindered performance.

Further analysis was focused on evaluating the validity of the fixation detection algorithm used in the proposed system. As will be discussed later in “Conclusion and discussion”, the current algorithm lacks sophistication, relying upon basic heuristics mostly derived from eye-tracking studies using head-fixed desktop setups on static objects, and therefore could produce errors in our setup using head-mounted eye-tracking during free navigation in the natural environment with dynamic objects. A particular concern was on the threshold value of 5.2° used to recognize whether, compared to the previous frame, the gaze is staying on the same object or has been shifted to a “different object of the same label” (DOSL hereafter), and how errors arising from these heuristics would affect the persistency value. To this end, a comparison between human coders and the system was done for frames where human coders marked “New Item?” and the frame where the system detected DOSL events.

First, it can be seen that the DOSL event is rare; DOSL occurred 34 times out of the total 3543 frames processed by the system. Of these times, the proposed algorithm successfully identified the end of fixation 16 times (47.06%). The remaining 18, termed false-negative fixation terminations, occurred because gaze moved to another instance of the same object class and deviated less than 5.2° from that observed in the previous frame. Notably, such errors commonly (86%) occurred when these objects were located close to each other in space (e.g., vehicles parked side-by-side), or, more rarely (14%), in response to objects being located far away from the eye-tracking device; in such cases, saccade size between object fixations was less than the 5.2° threshold. In addition, the algorithm also caused 15 false-positive fixation termination errors, i.e., the gaze moved beyond the 5.2° threshold while remaining on the same object. This suggests that the suggested heuristic algorithm is only marginally effective in detecting DOSL events.

Even though the accuracy of the proposed algorithm is found to be insufficient to capture all DOSL events, these errors had minimal impact on overall persistency scores due to the rarity of these events. The persistency scores for three representative labels, i.e., Person, Truck, and Car, before and after the correction of the errors (both false-negative and false-positive) for all videos are summarized in Table 3. Therefore, we conclude that, while future work should explore more sophisticated approaches (for possible future implementations, see  “Conclusion and discussion”), this simple heuristic was sufficient for such investigations as that presented in this paper.

**Table 3:** The change in average persistency scores before and after the correction procedure. Note that persistency is measured according to the number of consecutive *frames* in which each object was gazed upon

Object	Persistency score before correction (frames)	Persistency score after correction (frames)
Person	8.813 (SD = 1.075)	8.250 (SD = 0.829)
Truck	14.14 (SD = 0.532)	14.07 (SD = 0.440)
Car	15.508 (SD = 1.448)	14.016 (SD = 1.110)

**Fig. 6:**
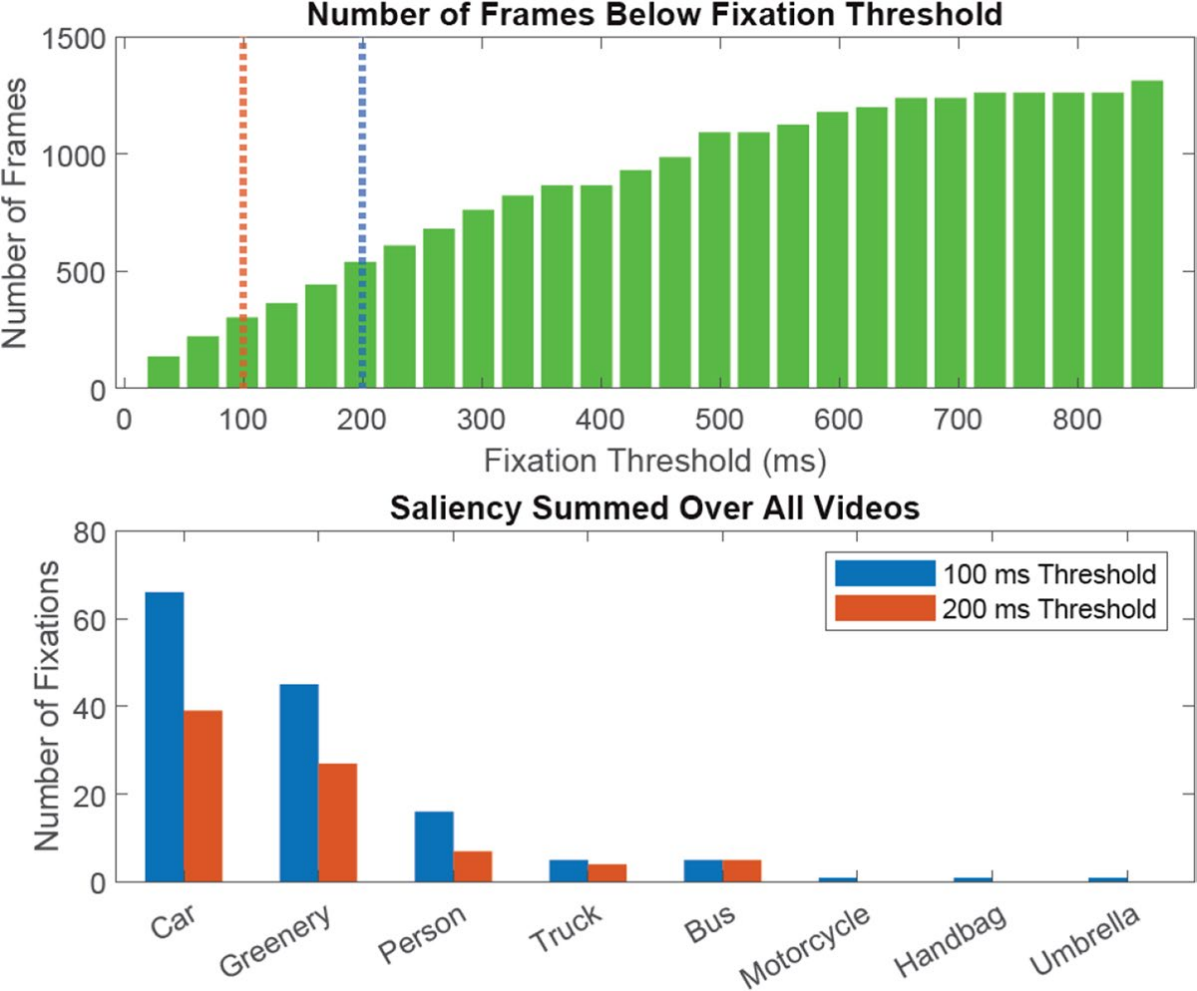
Top: Cumulative distribution of frames that belong to different fixation durations. Red and blue dotted lines indicate the 100 and 200 ms threshold respectively. Bottom: Number of fixations for object labels when different thresholds for fixation are applied

As described before, the proposed system determines whether the object is fixated or not by using a dwell time threshold of 200 ms. Below that threshold, the gaze is considered to be momentarily overlapping with the object, rather than actually fixated on it. Although the 200 ms threshold was adopted from a well-known study by Salvucci and Goldberg (2000), it should also be noted that the minimum dwell time for fixation varies significantly between 100 ms or more, depending on the scenario (Lappi, 2016). For this reason, additional verification was conducted to check how much the above-obtained scene variables, especially the saliency, vary depending on the choice of the fixation time threshold. Figure 6 summarizes the verification result. The first plot (Fig. 6 top) shows the cumulative distribution of the frames by different threshold values. Out of a total of 3543 frames (six videos of 20 seconds each recorded in 30 Hz) used for evaluation, a gaze was on a recognizable object for 1314 frames. Among those frames, 23.1% (304) were discarded by the 100 ms threshold, and 41.1% (540) were discarded by the 200 ms threshold. This indicates that there is a substantial number of frames (18%) whose dwell time was between 100 and 200 ms. However, the second plot (Fig. 6 bottom) suggests that, although there were expected increases in the number of fixations when the threshold changes to 100 ms, the overall trend of the saliency distribution is preserved. This suggests that the observed saliency scores may not be significantly affected by the choice of the fixation threshold.

**Fig. 7:**
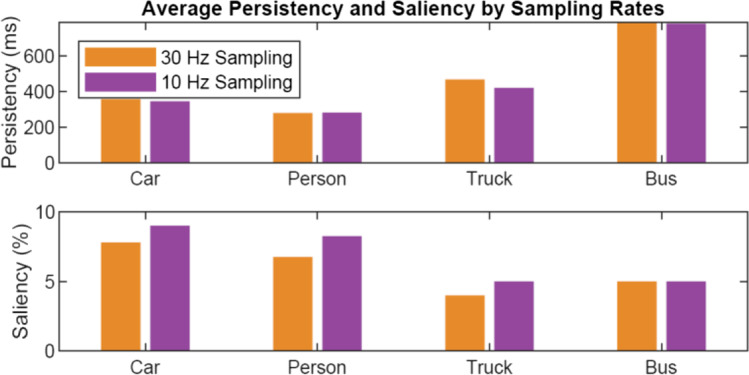
Comparison of gaze analysis results using 30 vs. 10 Hz sampling rate. Persistency and saliency of four representative object labels were computed for six video clips used for evaluation. Top: Average persistency scores. Bottom: Average saliency scores

In addition, it should be noted that, although the system evaluation was conducted by asking the human coders and the proposed system to annotate every frame of the 30 Hz scene video, the results of annotating the entire scene videos presented in the case study are based on analysis of one in every three frames (i.e., 10 Hz) due to limitations in computing power. This could potentially affect the scene variables that are reported later in the case study (Fig. 10). Therefore, additional analysis was conducted on video clips used for evaluation to check how much the gaze-related scene variables, i.e., persistency and saliency, are affected by down-sampling from 30 to 10 Hz. As can be seen from Fig. 7, it was found that both variables are barely affected by the down-sampling: the differences in persistency and saliency were all less than 50 ms and 1.5% respectively. Based on these results, it was concluded that the effect of down-sampling on the scene variables of the entire video footage was not substantial.

Lastly, as a major advantage of the proposed system, we also compare the amount of time spent in video processing for both human coders and the proposed system. The proposed system took an average of 274.16 seconds (4 minutes and 34 seconds) to annotate each 20-second video clip consisting of around 600 frames. Note that the Mask R-CNN was run on a free GPU provided by Google Colaboratory, which can be considered as a baseline performance platform for running deep neural nets. The processing speed will be significantly improved when high-performance GPUs are used. Nevertheless, the processing time was considerably faster than those reported for manual coders: On average, manual coders took 42 minutes to annotate a 20-second sample. It also should be noted that the time taken by the human coders would not linearly scale; annotating the entire 30-minute video with 18,000 frames would be virtually impossible for human coders.

## Case study

To assess the practicality of the proposed system, this case study focused on how applying our system to an existing study can lead to an improvement in the quality of the experimental method and the specificity of data analysis, compared to the conventional framework screen-based settings with no or manual annotation. Note that the purpose of the case study is purely to show such potential, not to propose a new scientific finding, which would require a much more rigorous experimental design with a substantially greater number of participants.

We applied the proposed system to a recent study by Toth et al. (2020) on attentional bias induced by prior exposure to different environments. In their original study, Toth et al. (2020) suggested that participants being previously exposed to an urban environment have greater difficulty in inhibiting the innate attentional shift towards emotional—specifically fearful—faces, while prior exposure to a natural environment does not have any effect. They explained that the multitude of distractions apparent in urban environments consume central cognitive resources, and therefore disrupt top-down inhibitory control (Pourtois et al., 2013), especially when inhibiting a stronger attentional shift towards fearful faces (Horstmann et al., 2006; Pourtois et al., 2004).

Importantly, Toth et al.’s (2020) study was based on the conventional lab-based experimental paradigm without eye-tracking and therefore, despite the convincing result, possesses several drawbacks in their experimental and analysis methods. First, the experiment required participants to passively observe a screen depicting a first-person video of a confederate navigating a route. As shown in previous studies, such as Kort et al. (2003), the psychological effect of in vivo versus virtual environmental exposure can be very different, even when highly immersive virtual reality devices are used. Therefore, switching to an in vivo environment would improve ecological validity and would reduce the probability of a type II error. Secondly, while exposure to a certain environment involves exposures to many elements constituting the environment and the behavioral effects which they induce, such as the number of attention-getting objects or resultant changes in gaze distributions, the study limits its investigation to a single factor, i.e., urban or nature. As with other studies with the conventional paradigm, these limitations are mainly due to the lack of tools for measurement and automatic analysis, and therefore incorporating mobile eye-trackers and the object detection and gaze annotation algorithms proposed by our system is expected to significantly improve the veridicality and specificity of the analysis.

For the above reasons, we incorporated the proposed system by having participants physically navigate two routes, one urban and one in nature, while wearing the mobile eye-tracking device. After initially assessing for the existence of the city-induced attentional bias towards fearful faces, we use the system’s output statistics to determine how the strength of the bias was influenced by gaze behaviors during navigation under different environments, exploring whether different types of objects being focused during navigation impact the strength of the attentional bias.

### Case study methods

#### Experimental design

**Fig. 8:**
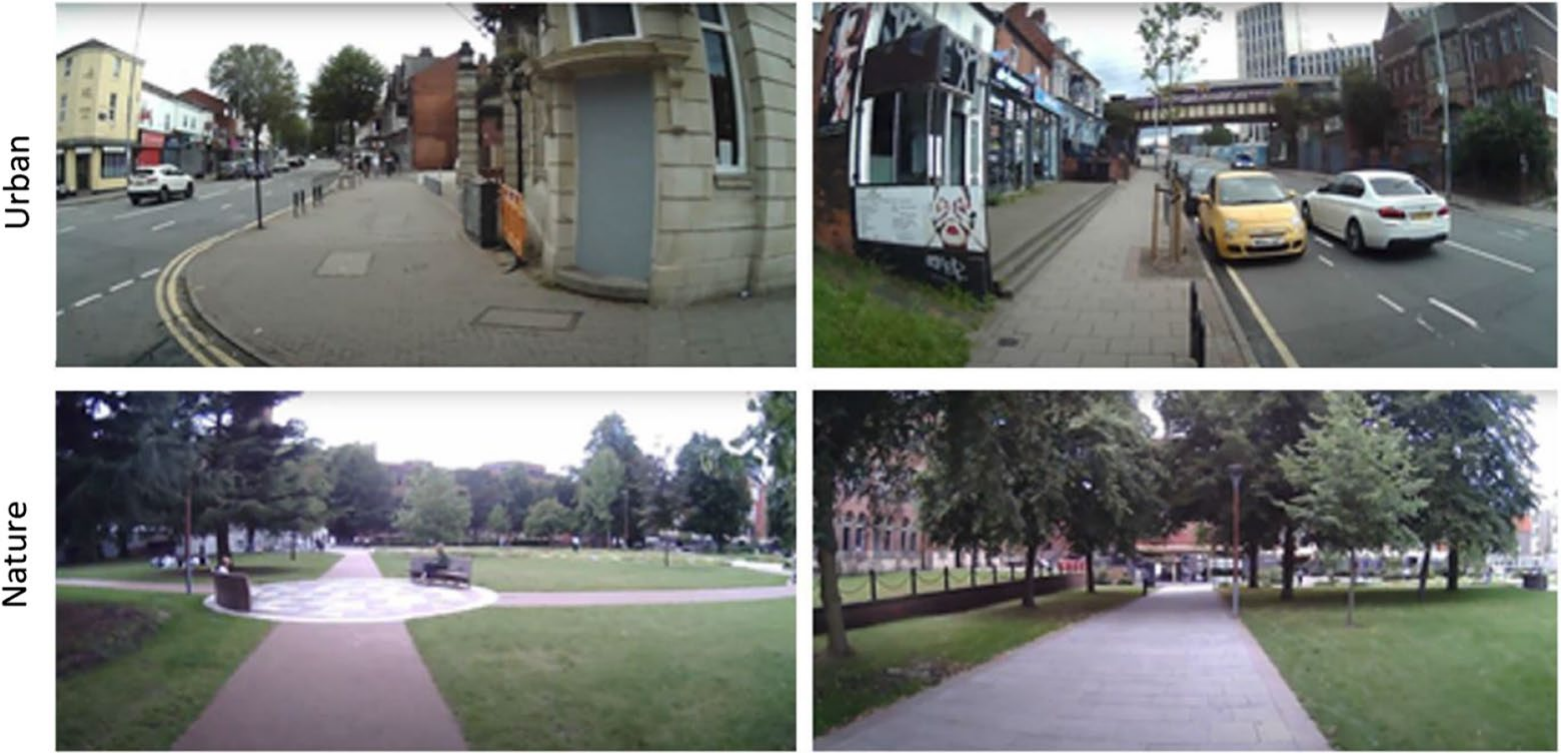
Screenshots from the urban (top) and nature (bottom) routes

Six naïve participants, aged between 18 and 30, completed two sessions, navigating one environment (nature or urban) in each. Due to technical difficulties with the eye-tracking device resulting in multiple frames of footage without gaze data readings, one participant’s data was not used in subsequent data analysis. In each session, participants were instructed to navigate one of the two environments via a prescribed route before completing a cognitive task designed to capture any environment-induced attentional bias. Routes were 1.25 miles long and took 25 minutes to complete with walking speed (3 mph) being dictated by an experimenter who accompanied the participant on their right-hand side. The urban course took participants to a city street mixed with driveways and sidewalks, while the nature route was within a parkland at the center of the university campus (Fig. 8). Sessions were separated by 1-week intervals and the order of the environmental exposures was counterbalanced across participants. Note that video samples taken from these trials were used for evaluation in “System evaluation”.

Before trials, participants completed a pre-trial calibration procedure. While keeping the head stationary, their gaze followed a target circle held by an experimenter standing two meters away. The experimenter moved this marker around while staying within the participant’s visual field and stopped at nine junctions to allow the eye-tracker’s calibration software to locate the marker and adjust calibration accordingly. Upon completion, the accuracy of tracking was ensured by instructing participants to fixate on various physical targets within the environment while the experimenter observed the screen to check for accurate gaze tracking.

Once completed, the laptop was packed into a backpack held on the participant’s back and the navigation began. The first five minutes of the trial were not recorded to give participants a chance to grow accustomed to the headset, thus ensuring natural gaze behaviors during the later (recorded) part of the trial. Once the remaining 20 minutes were complete, the calibration test was repeated to ensure that no mid-trial slippage had occurred. Notably, on occasion, the experimenter retested calibration *during* navigation when they believed that gaze tracking accuracy may have fallen, for example in response to significant changes in lighting conditions or when the device had been jogged. On all occasions, the experimenter judged the calibration accuracy to be sufficient for navigation to continue.

**Fig. 9:**
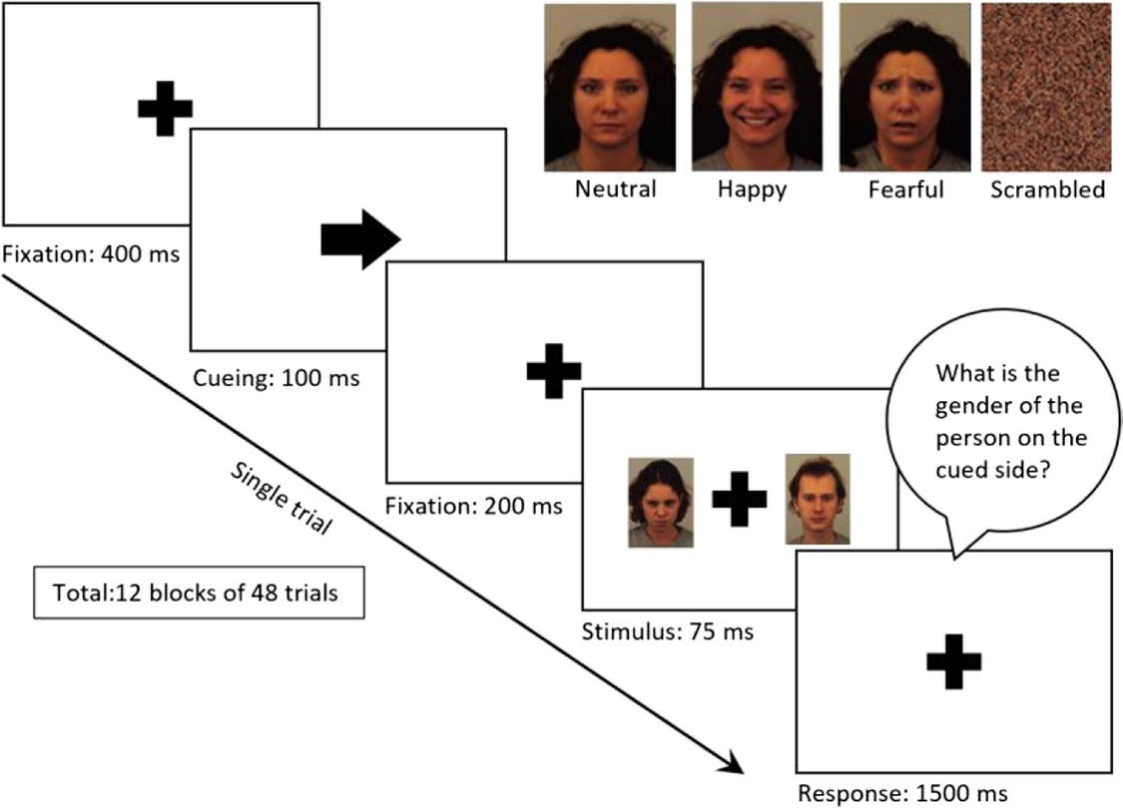
Experimental procedure for the cognitive task. Images on the upper-right corner show example stimuli: the target neutral-expression face (left), happy-face distractor (middle left), fearful-face distractor (middle right), and scrambled distractor (right)

Immediately following route completion, participants were seated in front of a computer screen and carried out a screen-based experiment adopted from Toth et al. (2020). The experiment was designed to measure the reaction time (RT) of recognizing the gender of a target facial image on the previously cued side paired with a distractor facial image portraying various emotions on the opposite side. For each trial, participants were first asked to fixate on a central cross for 400 ms. After that, a cue arrow, pointing either left or right, was displayed for 100 ms and then changed back to the fixation cross for another 1000–1500 ms. Up to this point, participants were instructed to maintain their fixation on the central markers. After that, images appeared on both sides of the screen for 75 ms. The image on the side that was previously cued by the arrow was always a neutral face, but the distractor image on the opposite side was either a neutral, happy, or fearful face, or a scrambled image. After that, all images disappeared except the fixation cross, and the participants were instructed to report as quickly as possible via keypress the gender of the face on the cued side with 1500 ms timeout. The human facial images were selected from the Karolinska Directed Emotional Faces data set (Lundqvist et al., 1998) and the experiment was programmed in MATLAB Psychtoolbox 3 (Brainard, 1997; Kleiner et al., 2007; Pelli, 1997). Figure 9 illustrates the overall experimental procedure.

The experiment consisted of 12 blocks of 48 trials, and the response time and accuracy for each trial were recorded for analysis. Attentional bias is reflected by the degree to which each type of distractor image (neutral, happy, fearful, and scrambled) interferes with task performance (reporting the gender of the target face), with slower RTs indicating greater interference (Fox et al., 2000; Hansen & Hansen, 1988). To measure bias, average RTs for each of the distractor types were compared to those of scrambled-face controls. As previous findings registered a bias towards fearful-face stimuli following exposure to urban environments, these trials were of particular interest: prolonged response times for trials with fearful-face distractors would indicate the existence of an attentional bias *towards* fearful stimuli.

#### Data processing and analysis

First, we compare scene and gaze statistics provided by the proposed system (i.e., saliency, frequency, and persistency). For simplicity, the analysis focused only on the three major features that typified the respective environments: Vehicles, Greenery, and Persons. Thus, system output was edited to only include these features; all occurrences of Car, Bus, Motorcycle, or Truck labels within the system’s GU-list were grouped into a common Vehicle label. All other recognized objects were given the general Other label. Frames in which no recognizable object was gazed upon were assigned the Background label, and frames with no associated gaze position (due to either intermittent eye-tracker failure or gaze moving beyond the bounds of the forward-facing camera) were tagged with an Out Of Bounds (OOB) label.

With this reduced GU-list, the system provides scene variables for each key feature. Saliency scores first present the percentage of frames in which gaze fell upon Vehicles, Persons, and Greenery. Frequencies for Vehicles and Person features compute the average number of vehicles and people appearing in the scene per frame. Note that Greenery frequency scores take the percentage of greenery pixels per frame averaged across all frames. Persistency scores then captured the average fixation length for all features using the heuristic algorithm outlined in “The complete gaze detection system”. These scene variables were computed for each participant and used for comparison between the two environments. In addition, since it is reasonable to assume that the frequency has a major effect on the saliency (i.e., objects that are more frequent in the environment are gazed at more often), we introduced an extra variable, *adjusted-saliency*, a Bayesian-like measure of saliency favoring objects that appeared less frequently, defined as saliency divided by frequency. This adjusted-saliency is expected to provide a rough estimation of which object, regardless of how frequently it appeared, drew more attention to the participants than others.

After scene and gaze characteristics were analyzed for nature and urban conditions respectively, they were then compared to the attentional bias measures, i.e., the reaction times of the cognitive task. Since the case study involved a limited number of participants and, more importantly, its goal is to show how the various analytical capabilities of the proposed system can provide insights into the exploration of various effects, the analyses only focused on providing descriptive statistics capturing overall trends observed in the data. The analysis was first focused on the main question of the original study by Toth et al. (2020) of whether navigations in nature versus urban environments had differential effects on attentional bias. Subsequent analyses were focused on exploring trends of how the major experimental variable related to attentional bias, i.e. RTs to fearful-face distractors, is affected by individual scene variables (frequency, saliency, and persistency) obtained by the system.

### Case study results

#### Scene and gaze characteristics

**Fig. 10:**
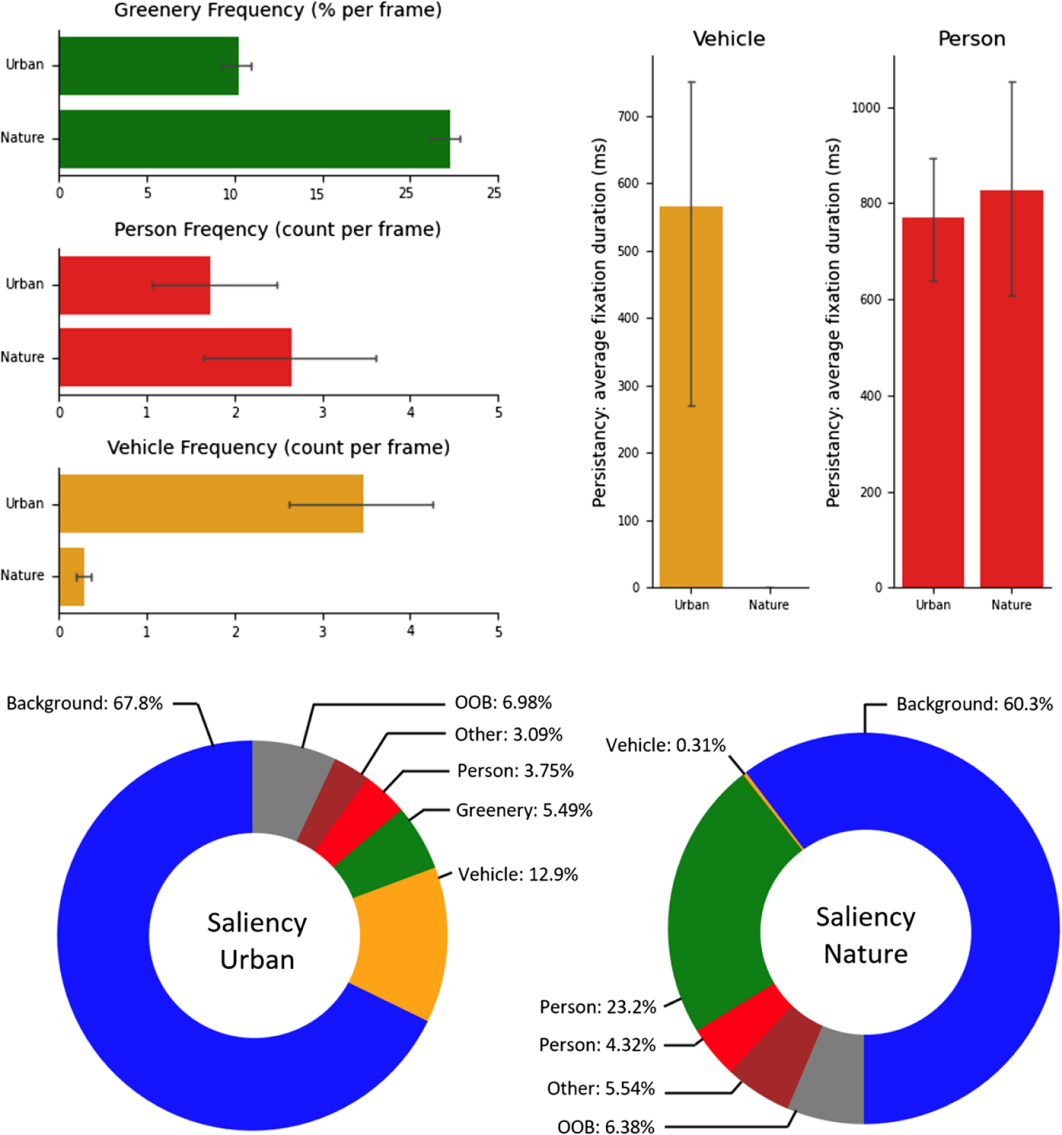
Scene variables computed. Top left: Bar charts presenting the frequency: greenery per frame (top), the number of people per frame (middle), and vehicles per frame (bottom) for city and nature trials. Top right: The persistency when a person (left) or vehicle (right) object was gazed upon. Bottom: A pie chart summarizing the saliency, i.e., the percentage of frames where each feature was gazed at, averaged across comparison samples for each environment

Figure 10 summarizes the output scene variables. The frequency computed for Greenery, Person, and Vehicle (Fig. 10, top-left) indicate that, as expected, participants encountered greenery objects more frequently in nature compared to urban environments. Rather unexpectedly, the frequency for Person class shows that the average number of people encountered during nature navigation was higher than that of urban navigation. Possible reasons for this and its potential impact on the result will be discussed later in “Conclusion and discussion”.

Persistency analysis (Fig. 10, top-right) showed that average fixation durations for people (Person label) were similar for both conditions (mean ± SD: 770.2 ± 137.8 ms for urban vs. 826.1 ± 286.4 ms for nature), while low frequency for the Vehicle in the nature condition rendered any corresponding persistency comparison uninformative.

The result of saliency analysis (Fig. 10, bottom) revealed expected trends: participants’ gazes were attracted more often by Vehicle objects during the urban condition (urban: 25.7% vs. nature: 0.34%) whereas they were attracted more often to Greenery objects during the nature condition (urban: 1.01% vs. nature: 39.9%). The following analysis on adjusted-saliency revealed that people (Person label) were gazed upon at a higher rate in nature (4.378) compared to the urban (2.559) environment. Similarly, green objects (Greenery label) were also gazed upon at a higher rate in the nature (1.773) than in the urban (0.135) condition. This suggests that participants paid greater attention to both people and greenery objects in nature compared to the urban environment.

#### Attentional bias analyses

To replicate the main analysis in the Toth et al. (2020) study, we compared RTs for fearful distractors after exposure to two different environments. Interestingly, this data does not seem to demonstrate evidence of the attentional bias observed in the original study: the mean reaction times for fearful faces do not seem greater following urban exposure (mean ± SD: 603.9 ± 98.7 ms) compared to nature exposure (599.8 ± 100.5 ms). As stated earlier, since the goal of this case study was solely to test and illustrate the capabilities of the proposed system, this case study focused only on speculating trends in the results without attempting quantitative comparisons, since proper confirmation of the trend may require a substantially greater number of samples.

**Fig. 11:**
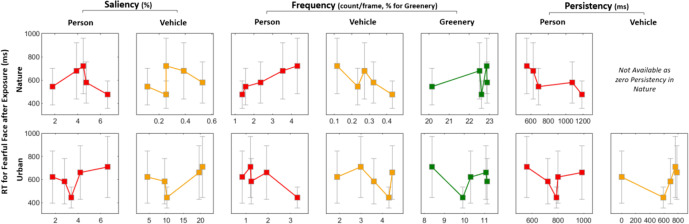
Visualizations of the relationships between RTs for fearful-face distractor trials and each of the three Scene variables. Each data point represents an individual participant

Subsequent analyses explored how the scene variables, namely saliency, frequency, and persistency, are related to the attentional bias—specifically, the RTs for fearful-face distractor trials. Figure 11 presents a series of plots that visualize such relationships. There seems to be a positive relationship between the frequency of Person labels and RTs with fearful distractors for the nature condition, although it needs to be checked with a greater number of participants whether the relationship is just a coincidence or not. This could indicate that participants who encountered more people while navigating the natural environment were more distracted by fearful distractors in the follow-up experiment. Since the frequency of Persons was unexpectedly higher in the nature versus urban condition, it can be speculated that the absence of the effect of environmental exposure on the attentional bias may be explained by this subsequent analysis indicating a sensitivity of RT to the humans. Further discussion can be found in “Conclusion and discussion”.

## Conclusion and discussion

In this paper, we have proposed a novel solution to the analysis of mobile eye-tracking data. The proposed method combines gaze behavior measured by mobile eye-tracking glasses with the output of a recent DL-based object recognition and segmentation algorithm (Mask R-CNN) and enables fully automatic annotations of regions and objects gazed upon during in-the-wild navigation trials.

### Conclusion of system evaluation

The proposed method offers several important advantages over existing solutions. Compared to the conventional computer vision algorithms adopted by previous studies, the DL-based algorithm incorporated in our system, Mask R-CNN, is capable of recognizing objects in a more flexible, human-like way. This flexible recognition includes recognizing objects in different types, in the same type but different shapes, and also with a dynamically variable shape such as humans and animals. In addition, whereas previous object detection algorithms only output a bounding box fitting to the object, potentially including many non-object pixels when detecting thin or highly concave objects (e.g., traffic light poles consisting of horizontal and vertical bars), Mask R-CNN outputs a mask outlining the exact region in the pixel space occupied by the object. This makes it an ideal choice for an algorithm for the accurate detection of whether an object is being gazed upon or not. Finally, the proposed method also provides freedom to define masks in different modalities, such as color-based masks (Greenery masks used in our case study), tailored to specific research questions.

In order to closely capture the characteristics of objects appearing in scenes, and that of the subject's gaze behavior, the proposed system computes three scene variables—frequency, saliency, and persistency—for each label recognized in the scenes. As exemplified in the case study, this allows for a more in-depth analysis scrutinizing potential latent interactions among scenes, gaze, and psychological effects. Running such analyses manually with human coders would be intractably time-consuming, especially when analyzing long and rich-in-objects video footage.

The evaluation of the proposed system focused on assessing the performance of the proposed system compared to human coders. First, we performed a thorough sanity check of a range of measures representing the annotation characteristics of human coders and of the proposed system: the variability among human coders, the commonalities of labels freely annotated by human coders, and the extent to which the recognizable labels of the proposed system cover the labels used by the human coders. As a result of this prior analysis, we found that the way human coders recognize and label objects is consistent with each other and also similar to the recognition ability of the proposed system. Although there were labels that are not recognizable by the proposed system, it was concluded that these labels had a minimal impact on the analysis since they occupy a small fraction of the data.

In the main analysis, the accuracy of the proposed method was verified by comparing the output of the proposed system with that of human coders. The similarity between the list of labels produced by human coders and the GU-list generated by the proposed system was measured using a standardized similarity measure, weighted average F1-scores. As indicated by overall high scores, the result suggested that the performance of the proposed system is comparable to that of human coders.

The post hoc analysis was particularly focused on the detection of DOSL events, of which the detection is done using a relatively crude heuristic algorithm based on eye-in-head movement. First of all, the analysis showed that the DOSL event occurs rarely, suggesting that the scene variables acquired from the navigation scenarios of the study, especially the persistency, are not heavily affected by the choice of the detection algorithm for DOSL. The comparison of DOSL events detected by human coders and those detected by the proposed system suggests that the performance of the heuristic algorithm of detecting the true-positive event is roughly on par with false positives and false negatives, indicating that the performance of the heuristic algorithm is rather limited. However, it is believed that the algorithm is still worthy of inclusion in the system since it can effectively prevent extreme false negatives when gaze highly deviates from one object to another. More fundamental solutions for DOSL detection that may be considered for future work will be discussed in “Limitations and future work”.

To ensure that the outputs of the system, especially the scene variables, are not significantly affected by the choice of data processing parameters, additional analyses compared the results based on the different choices of two parameters: fixation threshold (100 vs. 200 ms) and sampling rate (10 vs. 30 Hz). The results of both analyses suggest that, while some differences are observed, either persistency or saliency score, and their trends over different object labels were not sensitive to the choice of the processing parameters.

The performance difference between human coders and the proposed system becomes obvious when comparing the processing time and the scope of variables that can be extracted from each scene. This highlights the main advantage of the proposed method that, whereas the capacity of analysis is bottlenecked by the manual annotation procedure, our automatic recognition and annotation offers a scalable solution, both in terms of the feasibility of processing a large volume of data and also the diversity and specificity of analysis with a wide range of variables extracted from scenes.

### Conclusion of case study

The advantage of the proposed system in real research scenarios was exemplified by the presented exploratory case study replicating a previous screen-based experiment in a real navigation setup using the proposed system. We focused on a recent study by Toth et al. (2020) investigating the effect of exposure to a certain environment, urban or nature, to attentional bias toward threatening stimuli measured by the delay of RT induced by distraction from fearful faces. We replaced the video-based sessions used in Toth et al.’s (2020) study with de facto environmental exposures by asking participants to physically walk in urban or natural environments. The result of our analysis indicates that, unlike Toth et al.’s (2020) result, there is no obvious effect of exposure to different environments on the amount of distraction by fearful faces. Instead, further analyses looking for a relationship between the extracted scene variables and the RTs suggested that there could be a correlation between the amount of distraction to fearful faces and the number of people that participants encountered during their navigation in the natural environment.

The purpose of the exploratory case study was to exemplify the system’s potential, demonstrating how the method can be integrated into the existing study framework and can unlock new insights, and therefore we leave further verifications of the result to future work. However, our result suggests several points that warrant further investigation. First, an important difference between Toth et al.’s (2020) study and ours, apart from the video versus real navigation setup, was that participants encountered people during navigation in the nature environment in our setup, whereas the “nature” video used by Toth et al. (2020) did not include any persons in the view. In fact, since the nature environment chosen in our study was a park area in the university campus, the actual number of people encountered during the navigation was greater than that in the urban environment. Therefore, the most convincing explanation about the contradicting results between Toth et al.’s (2020) and our study would be that the attentional bias is primarily affected by the number of people encountered. This is also discussed by Toth et al. (2020), in that exposure to people in close proximity may have activated brain regions associated with threat-related attentional bias (Kennedy et al., 2009). The reason why such an effect was not observed in the urban condition is not clear; it may indicate a complex interaction between the cognitive effect and the types and frequencies of objects seen during navigation (Killgore & Yurgelun-Todd, 2005), which can only be explored further with a large amount of data with automatic scene analysis enabled by annotation systems such as ours.

As exemplified in the case study, the proposed method opens new possibilities in the field of gaze research by eliminating the time of manual annotation, which was one of the main limiting factors on the quantity of data such studies could handle. We expect that our method will permit so-called “big data” approaches (e.g., Wu et al., 2013) in gaze research in naturalistic scenarios, aiming to learn statistical relationships from a large volume of uncontrolled data, collected over hours and days. This is in sharp contrast with the conventional lab-based approaches using precisely controlled stimuli and accurately measured gaze profiles, but with a cost of highly limited data size. Considering the intrinsic uncertainty of the “gaze-in-wild” that is often affected by a host of extraneous variables and anomalous events, it is likely that the cognitive principles underlying complex coordination among gaze, environment, and motor control can be better elucidated by enough statistical power achieved by big data.

### Limitations and future work

Due to the nature of the data-intensive, machine-learning approach, the object recognition capability of the proposed model is mainly constrained by the database used for training. This contrasts with the traditional framework of feature-based algorithms, in which the capacity of the system is mainly constrained by the performance of the incorporated algorithm in flexibly detecting objects with various shapes and kinds. As described earlier, the Mask R-CNN used in this study was trained using the MS COCO data set consisting of images with 91 object labels, which covers objects generally appearing in everyday scenes including both indoor and outdoor objects (Lin et al., 2014). This indicates that the proposed system is generally applicable to everyday scenarios but may not be sufficient for scenarios that are either unusual or are very specific, which will therefore require the inclusion of a specific data set tailored to the target scenario. For instance, there exist databases specifically targeted at indoor objects (Bashiri et al., 2018; Damen et al., 2018; Ismail et al., 2020; Samani et al., 2021), which contain a wider variety of object types and a greater number of images for each object. Also, depending on the analysis scenarios, training can be tailored to specific situations or specific types of objects using open image databases widely available at present, such as groceries (Klasson et al., 2019), documents (Antonacopoulos et al., 2009), or objects in low lighting conditions (Loh & Chan, 2019). Although incorporating such databases for additional training is straightforward in the proposed framework, it should also be noted that only a few such data sets provide instance segmentation information required for the masking. Depending on the given annotation scenario, the system can possibly compromise with non-masking object detection networks that only output bounding boxes (Carion et al., 2020; Redmon et al., 2016; Ren et al., 2015).

As mentioned earlier, the Mask R-CNN's inability to recognize whether an instance of an object is the same as the one detected in the previous frame, i.e., the absence of tracking function, works as the main limitation of the system. In particular, such inability may affect the system’s estimation of persistency, i.e., the average amount of time each type of object was gazed upon, since it cannot detect DOSL events. Based on the comparison with human coders, it was found that the absence of tracking function only has a small effect on the persistency computation and, at least for our scenarios, a simple heuristic algorithm incorporated in the system was sufficient for our scenario in preventing obvious misdetections due to DOSL events. However, improvements in the following directions should be sought through future studies:

First, integration with a head-tracking function to the system can be considered to better classify the gaze behavior. It is important to point out that the proposed system records “eye-in-head” using a head-mounted scene camera and eye-tracker. During navigation scenarios, this is different from the “eye-in-space” that gaze behavioral studies are generally interested in since the participants can freely make body and head movements. Due to this inherent discrepancy, it is generally accepted that eye movement classification methods used in laboratory-based eye-tracking studies, where chin rests or bite bars are used to ensure no head movement, could cause ambiguities and misinterpretations when applied to free navigation scenarios (Lappi, 2016). For this reason, studies have shown that integrating a head-tracking function to the head-mounted system helps estimate the eye-in-space from eye-in-head movement. As a method for head tracking, conventional motion tracking devices including inertial measurement units (Kothari et al., 2020; Lanata et al., 2015; Larsson et al., 2014; Tomasi et al., 2016) can be integrated into the existing system. Alternatively, the algorithmic implementation of head movement estimation based on optic flow (Kinsman et al., 2012) can be also considered in future works, although such methods will have limitations in robustness and flexibility when processing dynamic scenes and varying shapes, compared to DL-based methods. The estimated eye-in-space movement will not only provide a more normative way to detect fixations without suffering from situations such as DOSL, but also will inform how the gaze interacts with the dynamic environment during natural navigation (Lappi, 2016).

Secondly, additional networks that enable tracking and other scene understandings can be integrated into the current Mask R-CNN network. Although relatively primitive, there have been continued efforts of implementing visual object tracking in R-CNN architecture (Voigtlaender et al., 2019; Voigtlaender et al., 2020), or non-object-based tracking based on image similarities (Steil et al., 2018). Incorporating such functionalities into the current system will provide a fundamental solution to spatiotemporally consistent annotation, such as correct detection of DOSL events.

In addition, another interesting direction for future development would be to integrate a monocular depth estimation network (see Ming et al., 2021 for a review). This will enable depth estimation for every pixel in the scene camera without requiring any additional sensors. Knowing the depth information of objects detected in the scene camera will bring an important advantage of more geometry-aware processing of gaze behavior into the analysis, such as the inclusion of distance or physical size of objects gazed upon.

Among object labels detected by the proposed system, the greatest portion was Background labels, which means that the gaze was not on any recognizable object. There are two possible cases that may have resulted in this labeling: (1) the gaze was actually on an object but the system was not able to recognize that object, and (2) the gaze was really not on any object. Based on the comparison of the system output with human coders, it can be confirmed that the occurrence of the first case is rare. As shown in the result, the performance of the proposed system in detecting objects in the scene was on par with that of average human coders, except for a few objects that are not included as object labels in the training data set. The second case is related to a rather philosophical view of defining what “objects” are. For example, one can premise that a visual scene consists of a seamless connection of objects, in which case everything, including sky, ground, and buildings, has to be included as objects. Alternatively, one can have a narrower view of the concept of an object and argue that a scene consists of objects sparsely located on a background defined as a broad term aggregating sky, ground, buildings, etc. The proposed system takes the latter point of view, and it can be seen through comparison with human coders that it is similar to the way humans recognize objects and the background. If the former view is required in future work, the system can alternatively implement semantic segmentation networks (see Asgari Taghanaki et al., 2021 for a review), which aims to partition a scene into semantic regions.

In addition, it should be noted that the recognition and annotation can be potentially affected by ambiguities arising from inherent hierarchies in object categories. For example, a vehicle contains different objects in its lower hierarchy, such as tires, windscreen, and side mirrors. Based on the scope of the annotation task or also the coder’s understanding, there can be variabilities in which object hierarchies are chosen and recognized. In our scenario, such ambiguity was not noticeable as shown by observed consistencies across human coders and also between human coders and the system. In fact, this observed similarity between humans and the system is rather expected since the data set used for training was also created by human coders. However, further consideration on the choice of training data set would be required when applying the system to special scenarios which demand recognition within specific object hierarchies.

As is evident in the presented results, while system output aligned well with that of human coders, there is also a degree of disagreement between the two. While additional system features and improvements in both object detection quality and fixation detection techniques would address this to a point, additional future work should explore the potential for a human-computer collaboration project. In such a case, the system output would act as preliminary results which the human can refine and correct. This mirrors similar work in the field of interactive machine learning, whereby model predictions generated with greater uncertainty are assessed and corrected by a human collaborator (Berg et al., 2019). Promising results in this field may generalize to similar objectives in eye-tracking research.

During our evaluation, care was taken to check whether the output of the proposed system was affected by the low spatiotemporal resolution of the system, especially the lower sampling frequency used for the entire scene video processing for computational efficiency. However, like all other eye-tracking studies, it is evident that the accuracy and precision of the presented system are reliant on the high spatiotemporal resolution of the eye-tracking device and the scene video, especially when processing objects that are far away and thus have small visual angles. Additionally, processing videos taken for hours and days, which is the ultimate usage scenario of the proposed system, would require significantly higher computational power compared to the one used in the current study. Therefore, future improvement of the system includes incorporating systems with higher spatiotemporal resolution head-mounted eye-tracking systems and higher computing power for video processing.

### Summary

In summary, we have proposed a system for automatic gaze annotation from eye-tracking data. The accuracy and the efficiency of the proposed system were validated by comparison with manual coders, and the potential of the system replacing conventional screen-based experiments was exemplified by the presented case study. The proposed system is expected to engender various future studies that combine the latest results of DL-based computer vision algorithms into the existing methods of experimental psychology, to enable a fully automatic and computationally intensive analysis of human behaviors.

## Data Availability

All data, code, and demonstration files can be accessed on the author’s website: https://github.com/OliDeane/Automatic_Gaze_Detection
